# Reproductive coercion in college health clinic patients: Risk factors, care seeking and perpetration

**DOI:** 10.1111/jan.15207

**Published:** 2022-04-01

**Authors:** Karen Trister Grace, Michele R. Decker, Charvonne N. Holliday, Janine Talis, Elizabeth Miller

**Affiliations:** 1 George Mason University, School of Nursing, Fairfax, Virginia, USA; 2 Johns Hopkins Bloomberg School of Public Health, Baltimore, Maryland, USA; 3 Division of Adolescent and Young Adult Medicine, UPMC Children's Hospital of Pittsburgh, Department of Pediatrics, University of Pittsburgh School of Medicine, Pittsburgh, Pennsylvania, USA

**Keywords:** college health, intimate partner violence, nursing, perpetration, reproductive coercion, sexual violence

## Abstract

**Aims:**

Reproductive coercion is associated with poor health outcomes in women. This study examined exposure to and use of reproductive coercion and care seeking among college students.

**Design:**

A cross-sectional survey was administered to 2291 college students of all genders seeking care in college health and counselling centres as baseline data for a cluster-randomized controlled trial.

**Methods:**

Online surveys were collected (9/2015–3/2017). Descriptive statistics, chi-square, Fisher's exact and *t*-tests were analysed.

**Results:**

Among female participants, 3.1% experienced reproductive coercion in the prior 4 months. Experience was associated with older age (*p* = .041), younger age at first intercourse (*p* = .004), Black/African American race (*p* < .001), behaviourally bisexual (*p* = .005), more lifetime sexual partners (*p* < .001) and ever pregnant (*p* = .010). Sexually transmitted infection (*p* < .001), recent drug use or smoking (*p* = .018; *p* = .001), requiring special health equipment (*p* = .049), poor school performance (*p* < .001) and all categories of violence (*p* = <.001–.015) were associated with women's reproductive coercion experience.

Participants who experienced reproductive coercion were more likely to seek care for both counselling and healthcare, (*p* = .022) and sexually transmitted infection (*p* = .004). Among males, 2.3% reported recent use of reproductive coercion; these participants reported sexual violence perpetration (*p* = .005), less condom use (*p* = .003) and more sexual partners than non-perpetrators (*p* < .001).

**Conclusion:**

Although reproductive coercion was reported infrequently among college students, those students experiencing it appear to be at risk for poor health and academic outcomes. Health and counselling centres are promising settings to address RC and related health behaviours.

## 1 ∣ INTRODUCTION

Reproductive coercion (RC) is a type of intimate partner violence (IPV) consisting of partner behaviours that coerce pregnancy through verbal pressure or birth control sabotage or controlling the outcome of a pregnancy by coercing or preventing abortion (Miller et al., 2010). Violence is pervasive among women aged 18–24 (Smith et al., 2018), but RC studies have largely focused on community-based or clinic-based samples of adult women. Limited studies of RC prevalence among younger women show that RC is prevalent, but factors that influence RC use and victimization among this population are unclear. In one study of 354 female college students who were also IPV survivors, 24.3% experienced RC in the past 6 months, and RC was associated with greater severity of abuse, relationship instability, missing class due to relationship problems and depression (Grace et al., 2020). A study of 146 female college students found that 25.3% had a partner who interfered with condom use (i.e., refusing to use one, removing it or tampering with it) in their lifetime (Katz & Sutherland, 2017). Another study of 233 female college students found a 29.6% prevalence of lifetime RC and a strong association between RC and IPV, as well as an association between RC and number of partners and decreased contraceptive and sexual self-efficacy (Katz et al., 2017). A study of 972 female college students found a RC prevalence of 8% (time frame not reported) and associations with IPV, history of pregnancy as well as unintended pregnancy and abortion, living with partner and Hispanic ethnicity (Sutherland et al., 2015). A study of 431 cisgender college students found that 13.9% of women reported past year RC and found associations with IPV, polyvictimization, sexual harassment and assault and younger age (no difference in the type of housing) (Swan et al., 2020). Taken together, these studies among college students highlight that RC is prevalent in this population, and that RC appears to be associated with IPV and poor reproductive health outcomes, consistent with the RC literature more broadly. Studies with college students are limited, however, by the use of abbreviated measures or a primary focus on condom interference. Engagement with college-age participants may reveal unique characteristics associated with violence and related health outcomes.

### 1.1 ∣ Background

Numerous studies have shown that the prevalence of IPV among patients seeking care in health settings is higher than the general population, probably related to the serious physical and behavioural health consequences of exposure to such trauma. Few studies have examined care-seeking behaviours of women who experience RC. One study of college students who had experienced IPV found that a larger proportion of women who experienced RC had sought help from a health-care provider for contraception, though this difference was not significant (Grace et al., 2020). In a community sample of women seeking care at family planning clinics, RC was associated with seeking pregnancy or STI testing and emergency contraception (Kazmerski et al., 2015). However, another study among high school students found no significant differences in pregnancy or STI testing/treatment or school health clinic reproductive health visits based on the experience of RC (Hill et al., 2019). RC was also not associated with seeking contraceptive services among female drug users (Perry et al., 2018). Assessment of care-seeking practices may elucidate characteristic patterns consistent with exposure to RC, to inform prevention and intervention by health-care providers.

Fewer studies have examined the use of RC in interpersonal relationships, i.e., RC perpetration. Quantitative research has examined men as victims of RC (Basile et al., 2019; Nemeth et al., 2019; Willie, Alexander, et al., 2019; Wright et al., 2018), but to our knowledge, only two studies have examined factors associated with RC perpetration, and none have studied college students specifically; one early study examined factors associated with IPV perpetration and found an association with preventing or pressuring abortion (Silverman et al., 2010) and one study of young couples examined RC victimization as well as perpetration (Willie, Powell, et al., 2019). Three qualitative studies with men examined motivations behind RC use (Alexander et al., 2019; Hamm et al., 2018; Thaller, 2017), and another described specific tactics of RC reported by men (Davis et al., 2014).

Emerging research documents that men also experience RC, but the magnitude of the abuse and health outcomes are unknown. However, a long-standing body of IPV research demonstrates gendered differences in the impact of partner violence. Violence victimization among women is more severe and more likely to result in a negative health impact, relative to IPV among men (Black et al., 2011). The original studies on RC examined associations with pregnancies that were mistimed, unplanned or unwanted (i.e., unintended pregnancies). For this study, we examine experiences of RC among people who are at risk for pregnancy (i.e., cisgender women and transgender men). For use of RC, we focus on individuals who generally are able to impregnate someone, i.e., cisgender men.

## 2 ∣ THE STUDY

### 2.1 ∣ Aims

The aim of this study was to examine correlates of RC victimization and perpetration as well as care-seeking behaviours in college students seeking healthcare and to identify behavioural factors and health outcomes associated with RC.

### 2.2 ∣ Design

Baseline data from a longitudinal intervention study were analysed. Study design and methodology are described in detail elsewhere (Abebe et al., 2018).

### 2.3 ∣ Participants

A convenience sample of 2291 students seeking care at 28 campus health centres (CHCs) on urban, rural and suburban college campuses in Pennsylvania and West Virginia were recruited. Students were eligible if they were 18–24 years old, enrolled at the school and had an appointment or were seeking care. The sample size was determined by power analysis for the parent study, a cluster-randomized control trial, and is described elsewhere (Abebe et al., 2018). Complete RC data were not present for 503 participants, making the final sample 1788 participants.

### 2.4 ∣ Data collection

Students completed online baseline surveys prior to their CHC appointment, between September 2015 and March 2017. Potential participants were identified by CHC staff at registration and informed about the study via flyers. If interested, they were assessed for eligibility by trained research assistants, given an information sheet about the study and provided oral or written signed consent (based on individual Institutional Review Board [IRB] requirements, see below).

### 2.5 ∣ Ethical considerations

Research procedures were approved by the primary research university's Human Subjects Research Protection Office as well as each college IRB. Resources were available on-site and embedded in surveys for participants who experienced emotional distress, safety concerns or alcohol/substance use.

### 2.6 ∣ Data analysis

Descriptive statistics were used to describe the study sample. Chi-square or Fisher's exact and independent sample *t*-tests were used to analyse differences between female participants who had and had not experienced RC on covariates and categories of care seeking and male participants who had and had not perpetrated RC on covariates. Sample size floated to accommodate small amounts of missing data. Analyses were conducted in Stata Statistical Software (StataCorp, 2019).

### 2.7 ∣ Measures

Measures included single items on demographics (including sex assigned at birth as well as gender identity), health history, educational status, reason for clinic visit, as well as the following:

*Sexual violence victimization* was assessed with six items adapted from other studies (Carey et al., 2015), which assessed the frequency of unwanted sexual experiences in the past 4 months. Experiences include ‘has anyone fondled, kissed or touched you sexually when you indicated that you didn't want to?’ and ‘has anyone made you have vaginal sex when you indicated you didn't want to?’ and one additional item assessing incapacitated multi-contact sexual experiences (‘was there an occasion when a group of individuals had sex with you against your will or when you were incapacitated (e.g., by drugs or alcohol) and unable to object or consent?’). Any unwanted sexual experiences greater than zero were considered any sexual violence exposure.

*Stalking* by an intimate partner was assessed with one item (Fisher et al., 2002): ‘In the past 4 months, how many times has a partner (or ex-partner) repeatedly followed you, watched you, phoned, written, e-mailed or communicated with you in other ways in a way that seemed obsessive and made you afraid or concerned for your safety? Any response greater than zero was considered stalking exposure.

*IPV* was assessed with one item from the Conflict Tactics Scale (Straus et al., 1996): In the past 4 months, how many times did someone you were dating or going out with physically hurt you on purpose? (Include such things as being hit, slammed into something or injured with an object or weapon.) Any response greater than zero was considered IPV exposure.

*RC* was assessed with four items from the abbreviated RC Scale (McCauley et al., 2017). Items included ‘In the past 4 months, has someone you were dating or going out with, or hooking up with: Told you not to use any birth control (like the pill, shot, ring, etc.) so that you would get pregnant; Taken your birth control (like pills) away from you or kept you from going to the clinic to get birth control so that you would get pregnant; Taken off the condom while you were having sex, so you would get pregnant; Put holes in the condom or broken the condom on purpose so you would get pregnant?’ and any affirmative response was coded as exposure to RC.

*Cyber dating abuse* was assessed with eight items (Dick et al., 2014) including such questions as: ‘In the past 4 months, how many times has a partner: made mean or hurtful comments to you using mobile apps, social networks, texts or other digital communication?’; ‘…spread rumours about you using mobile apps, social networks, texts or other digital communication?’; ‘…tried to get you to talk about sex when you did not want to using mobile apps, social networks, texts or other digital communication?’ Any cyber dating abuse experiences greater than zero was considered any cyber dating abuse exposure.

*Perpetration* measures included adapted versions of the above measures, for example, ‘In the past 4 months, have you done this to someone you were having sex with…’.

*Alcohol and drug use*, measures asked about frequency and amount of alcohol and drug use in the past 12 months (National Institute on Alcohol Abuse and Alcoholism [NIAAA], n.d.). Binge drinking was defined for assigned female sex at birth (AFAB) participants as four or more drinks in 2 h in the past 12 months, more than once a month, and for assigned male sex at birth (AMAB) participants as five or more drinks in 2 h in the past 12 months, more than once a month. Drug use included any use of all categories of drugs including marijuana.

*Sexual communication* was measured with four items asking how often, in the past 4 months, you discussed things like how to prevent pregnancy or how to use a condom with the person or persons you are having sex with (Edison et al., 2021; Milhausen et al., 2008). Any response greater than ‘never’ was considered the presence of sexual communication.

## 3 ∣ RESULTS/FINDINGS

The sample consisted of 1788 participants with an average age of 20.11 years. About 73.3% (*n* = 1308) of participants were AFAB and 26.8 % (*n* = 477) were AMAB. Of AFAB participants, 12 identified as male, transgender male, non-binary or none, and of AMAB participants, one identified as a transgender female.

### 3.1 ∣ Correlates of experiencing RC among AFAB participants

Only AFAB participants received the RC questions. About 3.1% of these participants experienced RC in the prior 4 months (Table 1). Participants who experienced RC were significantly older than those who did not (*p* = .041); this age difference was small (6 months), but in a college-age sample could reflect as much as one school-year difference. They also were significantly younger at age of first intercourse (close to 1 year; *p* = .004) and had significantly more lifetime sexual partners (9.42 vs. 5.14, *p* < .001). Other factors significantly associated with experiencing RC included Black/African American race (*p* = .001), being behaviourally bisexual (*p* = .005), ever having been pregnant (*p* = .010) or having an STI (*p* < .001), use of any drugs and smoking in past 30 days (*p* = .018; *p* = .001) and having a health problem that requires special equipment (*p* = .049). They were also more likely to be earning lower grades (C average; *p* < .001). All categories of violence in the past 4 months were significantly associated with experiencing RC: sexual violence (*p* = .015), IPV (*p* < .001), stalking (*p* = .001) and cyber dating abuse (*p* = .005).

### 3.2 ∣ Care seeking among AFAB participants who experienced RC

Participants who experienced RC were more likely to be visiting the college health centre for both counselling and healthcare, as opposed to either one or the other (*p* = .022; Table 2), and more likely to be seeking STI testing or treatment (*p* = .004). No significant difference was found for women attending the clinic for birth control or emergency contraception (*p* = 1.000) or for pregnancy testing (*p* = .374).

### 3.3 ∣ Correlates of RC perpetration among AMAB participants

A small subset of AMAB participants reported perpetrating RC (2.3%; Table 3). Correlation findings are limited by this small number, but perpetrators were more likely to report never or hardly ever using condoms over the past 4 months (*p* = .003) and to also report both lifetime (*p* = .005) and past 4 months (*p* = .026) sexual violence perpetration. RC perpetration was not associated with binge drinking, smoking, drug use, military status or athletic team or Greek life membership. While age and age at first intercourse were not risk factors, perpetrators did report significantly more lifetime sexual partners than non-perpetrators (17.80 vs. 6.74, *p* < .001).

## 4 ∣ DISCUSSION

This study contributes to the emerging literature on RC among young adults, specifically college students. Notably, this study highlights some previously not described correlates for RC victimization including drug use and smoking in the past 30 days and having a health problem that requires special equipment. The prevalence of RC was lower than other studies which only looked at IPV survivors (Grace et al., 2020) or which asked about RC experiences during a longer time frame (Katz et al., 2017; Katz & Sutherland, 2017; Swan et al., 2020) but consistent with other studies with general populations of college students (Sutherland et al., 2015). In addition to finding strong associations with IPV, consistent with previous studies, this study also expanded exposure to violence to include other forms such as stalking and cyber-dating abuse.

Relevant to concerns on college campuses about predictors of dropping out of college, RC was clearly associated with poor school performance as well as number of sexual partners and ever having been pregnant. Also consistent with the literature, RC in this sample of college students was associated with experiencing sexual assault (Basile et al., 2018), Black/African American race (Holliday et al., 2017), being behaviourally bisexual (Alexander et al., 2016), having an STI (Northridge et al., 2017) and age of sexual initiation (Fasula et al., 2018). Findings about race as a risk factor should be interpreted through a lens of critical race theory as well as other studies which identify structural factors of incarceration, housing and employment instability, which are disproportionately experienced by Black men, as potential motivating factors in RC perpetration (Nikolajski et al., 2015).

No significant difference was found for women attending the clinic for birth control or emergency contraception or for pregnancy testing given RC experience, contrary to previous research findings which found that experiencing RC was associated with increased odds of seeking pregnancy or STI testing, as well as emergency contraception in women up to age 30 (Kazmerski et al., 2015). This population of college students may be more highly motivated to avoid pregnancy, and some religious-affiliated schools were included, which may also have influenced this finding. Pregnancy tests, emergency contraception and condoms are also available over the counter without seeing a provider, which may also have impacted these findings. However, attending the health clinic for both physical and mental health was associated with RC, indicating a need for comprehensive and holistic care and pointing to the potential influences of RC on mental health.

More research is needed to understand factors associated with men's use of RC. To date, the studies focused on use of RC by male-identified individuals have recruited predominantly low-income men from community centres and health clinics, (Alexander et al., 2019; Davis et al., 2014; Dimenstein et al., 2021; Hamm et al., 2018; Silverman et al., 2010), or abuser intervention programs (Holliday et al., 2018), and most works in this area have been qualitative (Alexander et al., 2019; Davis et al., 2014; Hamm et al., 2018; Thaller, 2017). This study presents novel findings on correlates of RC perpetration among male college students, which included condom non-use, sexual assault perpetration and a substantially higher number of lifetime sexual partners. These findings highlight the association of RC with other sexual behaviours associated with poor health outcomes, including the use of sexual violence, suggesting that greater attention to discussing sexual consent, sexual violence prevention and condom use should be more explicitly integrated into sexual health education prior to matriculation on college campuses. Similarly, as far too many college students start higher education without sufficient comprehensive sexual health education in their middle and high school years (Lindberg & Kantor, 2021), greater attention to integrating sexual health education directly into sexual violence prevention education is clearly needed.

### 4.1 ∣ Limitations

Findings should be considered in light of several limitations. The study relied on self-report of sensitive topics; however, surveys were administered online or in private areas to improve privacy. Schools included are not representative of all college students across the country, and racial/ethnic groups as well as gender and sexual minorities were underrepresented in the sample. Additionally, the number of AMAB participants in this sample and the prevalence of RC perpetration in this group were small, precluding adjusted analyses and limiting conclusions that can be drawn about correlates of RC perpetration. Likewise, AMAB participants may have been reluctant to reveal RC behaviours and other perpetration behaviours such as sexual assault, despite the anonymity of a tablet-based survey. Future research should recruit a larger sample of AMAB participants and consider other methods of assuring participant confidentiality. The correlation of RC experience with health problems requiring special equipment and emerging qualitative research on RC in AFAB people with disabilities (Alhusen et al., 2019) points to an area of RC study which we know little about, but which warrants further examination. The correlation between RC and smoking among both perpetrators and survivors should be examined further.

## 5 ∣ CONCLUSION

This study's novel findings inform developing knowledge about a critical form of coercion in relationships. Although the lack of association between contraception needs and RC contradicts prior research, the finding that RC is associated with a range of other concerns such as academic performance, substance use and STI testing and treatment all highlight the need for health professionals serving AFAB college students on campuses to be aware of the possibility of RC among students seeking care in student health and counselling centres, especially students who can become pregnant. RC is associated with other forms of interpersonal violence and sexual behaviours, underscoring the need for integration of sexual health education into college campus sexual violence prevention efforts. As with other clinical settings such as family planning and adolescent health clinics, AFAB college students seeking care in campus health centres may be experiencing RC and health professionals should consider the routine provision of education about RC and supports and services available (including harm reduction strategies) to college students.

## Figures and Tables

**TABLE 1:** Demographics and correlates of experiencing reproductive coercion among participants assigned female at birth (*N* = 1308)

Characteristic	Full sample *N* (%)	Experienced RC *n* (%)	Did not experience RC *n* (%)	*p*-value^a^
Experienced any RC behaviour (past 4 months)				
No	1268 (96.9)			
Yes	40 (3.1)			
*Age—mean (SD),n= 1293*	*20.10 (1.52)*	*20.59 (1.76)*	*20.08 (1.51)*	***.041***
*Age at 1st intercourse—mean (SD), n = 1247*	*16.90 (1.94)*	*16.03 (1.64)*	*16.93 (1.95)*	***.004***
*Lifetime sexual partners—mean (SD), n = 1204*	*5.27 (6.01)*	*9.42 (11.90)*	*5.14 (5.68)*	***<.001***
*Self-rated SES (scale of 0–100)—mean (SD), n = 1271*	*38.49 (17.54)*	*40.62 (17.95)*	*38.43 (17.54)*	*.454*
Hispanic/Latinx				.604
No	1137 (87.0)	35 (89.7)	1102 (86.9)	
Yes	170 (13.0)	4 (10.3)	166 (13.1)	
Race				**.001**
American Indian or Alaska Native	5 (0.4)	0	5 (0.4)	
Asian	51 (3.9)	0	51 (4.0)	
Black or African American	134 (10.3)	15 (37.5)	119 (9.4)	
Native Hawaiian/other Pacific Island	2 (0.2)	0	2 (0.2)	
White	1032 (79.0)	24 (60.0)	1008 (79.6)	
Multiracial/More than one race	61 (4.7)	1 (2.5)	60 (4.7)	
Other	21 (1.6)	0	21 (1.7)	
Behaviourally bisexual				**.005**
No	1147 (91.5)	31 (77.5)	1116 (92.0)	
Yes	106 (8.5)	9 (22.5)	97 (8.0)	
Ever been pregnant				**.010**
No	1217 (96.4)	33 (86.8)	1184 (96.7)	
Yes	46 (3.6)	5 (13.2)	41 (3.4)	
Ever diagnosed with an STI				**<.001**
No	1181 (90.5)	28 (70.0)	1153 (91.2)	
Yes	124 (9.5)	12 (30.0)	112 (8.9)	
Condom use frequency, past 4 months				.284
Never or hardly ever	406 (35.5)	17 (43.6)	389 (35.2)	
Sometimes, almost all the time or every time	737 (64.5)	22 (56.4)	715 (64.8)	
Contraceptive use at last sex (other than condoms)				.367
None	124 (10.6)	5 (15.2)	119 (10.5)	
Pills/ring	499 (42.8)	14 (42.4)	485 (42.8)	
Injectable	39 (3.3)	1 (3.0)	38 (3.4)	
Implant/IUD	83 (7.1)	4 (12.1)	79 (7.0)	
Withdrawal	380 (32.6)	7 (21.2)	373 (32.9)	
Other	41 (3.5)	2 (6.1)	39 (3.4)	
Experienced sexual violence, past 4 months				**.015**
No	1097 (83.9)	28 (70.0)	1069 (84.3)	
Yes	211 (16.1)	12 (30.0)	199 (15.7)	
Experienced IPV, past 4 months				**<.001**
No	1271 (97.2)	33 (82.5)	1238 (97.6)	
Yes	37 (2.8)	7 (17.5)	30 (2.4)	
Stalking, past 4 months				**.001**
No	1226 (93.7)	31 (77.5)	1195 (94.2)	
Yes	82 (6.3)	9 (22.5)	73 (5.8)	
Cyber dating abuse, past 4 months				**.005**
No	803 (61.4)	16 (40.0)	787 (62.1)	
Yes	505 (38.6)	24 (60.0)	481 (37.9)	
Binge drinking				.161
No	666 (50.9)	16 (40.0)	650 (51.3)	
Yes	642 (49.1)	24 (60.0)	618 (48.7)	
Drug use, past 30 days				**.018**
No	925 (71.6)	22 (55.0)	903 (72.1)	
Yes	367 (28.4)	18 (45.0)	349 (27.9)	
Smoking, past 30 days				**.001**
No	1069 (82.5)	25 (62.5)	1044 (83.1)	
Yes	227 (17.5)	15 (37.5)	212 (16.9)	
Health problem requiring special equipment				**.049**
No	1228 (96.4)	35 (89.7)	1193 (96.6)	
Yes	46 (3.6)	4 (10.3)	42 (3.4)	
Any mental health disorder (Depression, anxiety, PTSD, bipolar, OCD, schizophrenia)				.745
No	556 (42.5)	16 (40.0)	540 (42.6)	
Yes	752 (57.5)	24 (60.0)	728 (57.4)	
Year in school				.178
1st year undergraduate	334 (25.5)	8 (20.0)	326(25.7)	
2nd year undergraduate	315 (24.1)	12 (30.0)	303 (23.9)	
3rd year undergraduate	300 (22.9)	7 (17.5)	293 (23.1)	
4th year undergraduate	226 (17.3)	8 (20.0)	218 (17.2)	
5th year undergraduate	39 (3.0)	4 (10.0)	35 (2.8)	
Graduate or professional	91 (7.0)	1 (2.5)	90 (7.1)	
Other	3 (0.2)	0	3 (0.2)	
Current residence				.523
Campus residence hall	642 (49.1)	15 (37.5)	627 (49.5)	
Fraternity or sorority house	15 (1.2)	0	15 (1.2)	
Other college/university housing	106 (8.1)	5 (12.5)	101 (8.0)	
Parent/guardian's home	51 (3.9)	1 (2.5)	50 (4.0)	
Other off-campus housing	483 (37.0)	19 (47.5)	464 (36.6)	
Other	10 (0.8)	0	10 (0.8)	
Average grades				**<.001**
A	530 (40.9)	9 (23.1)	521 (41.5)	
B	630 (48.7)	15 (38.5)	615 (49.0)	
C	131 (10.1)	15 (38.5)	116 (9.2)	
D/F	4 (0.3)	0	4 (0.3)	
Sexual communication				.832
No	328 (26.0)	11 (27.5)	317 (26.0)	
Yes	931 (74.0)	29 (72.5)	902 (74.0)	

Italics indicate *mean/SD*. Bold indicates significant at *p* < .05.

a By *t*-test, Chi-square or Fisher's exact test.

**TABLE 2:** Care seeking as a correlate of experiencing reproductive coercion among participants assigned female at birth (*N* = 1308)

Characteristic	Full sample *N* (%)	Experienced RC *n* (%)	Did not experience RC *n* (%)	*p*-value^a^
Type of visit to health centre				**.022**
Counselling	142 (11.8)	4 (12.1)	138 (11.8)	
Health appointment	1026 (85.2)	25 (75.8)	1001 (85.4)	
Both	37 (3.1)	4 (12.1)	33 (2.8)	
Reason for visit to health centre				
Cold/flu/allergy	303 (23.2)	11 (27.5)	292 (23.0)	.509
GI	28 (2.1)	0	28 (2.2)	1.000
Fatigue, fever, other pain	85 (6.5)	2 (5.0)	83 (6.6)	1.000
Physical	55 (4.2)	1 (2.5)	54 (4.3)	1.000
TB test	138 (10.6)	3 (7.5)	135 (10.7)	.792
Immunization	69 (5.3)	0	69 (5.4)	.266
Mental health	97 (7.4)	5 (12.5)	92 (7.3)	.213
Birth control or EC	90 (6.9)	2 (5.0)	88 (6.9)	1.000
Pregnancy test	15 (1.1)	1 (2.5)	14 (1.1)	.374
STI test or treatment	51 (3.9)	6 (15.0)	45 (3.6)	**.004**
Gyn exam	51 (3.9)	1 (2.5)	50 (3.9)	1.000
Genitourinary	39 (3.0)	1 (2.5)	38 (3.0)	1.000
Injury	34 (2.6)	0	34 (2.7)	.622
Sexual assault	5 (0.4)	0	5 (0.4)	1.000
Alcohol or drug counselling	15 (1.2)	0	15 (1.2)	1.000
Other	317 (24.2)	7 (17.5)	310 (24.5)	.313

a By Chi-square or Fisher's exact test.

**TABLE 3:** Correlates of reproductive coercion perpetration among students assigned male at birth (*n* = 477)

Characteristic	Full sample *N* (%)	Perpetrated RC *n* (%)	Did not perpetrate RC *n* (%)	*p*-value^a^
Used any RC behaviours, past 4 months				
No	466 (97.7)			
Yes	11 (2.3)			
*Age (age)—mean (SD), N = 468*	*20.13 (1.58)*	*20.82 (2.14)*	*20.11 (1.56)*	*.143*
*Age at 1st intercourse—mean (SD), N = 396*	*16.65 (1.95)*	*16.27 (2.57)*	*16.66 (1.94)*	*.513*
*Lifetime sexual partners—mean (SD), N = 387*	*7.02 (9.28)*	*17.80 (18.12)*	*6.74 (8.80)*	***<.001***
*Self-rated SES (scale of 0–100)—mean (SD), N = 458*	*37.82 (17.92)*	*42.18 (25.68)*	*37.72 (17.72)*	*.415*
Hispanic/Latinx				.698
No	393 (82.6)	10 (90.9)	383 (82.4)	
Yes	83 (17.4)	1 (9.1)	82 (17.6)	
Race				.050
American Indian or Alaska Native	2 (0.4)	0	2 (0.4)	
Asian	28 (5.9)	3 (27.3)	25 (5.4)	
Black or African American	78 (16.4)	2 (18.2)	76 (16.3)	
Native Hawaiian/other Pacific Island	0	0	0	
White	344 (72.1)	5 (45.5)	339 (72.8)	
Multiracial/more than one race	15 (3.1)	1 (9.1)	14 (3.0)	
Other	10 (2.1)	0	10 (2.2)	
Ever gotten someone pregnant				.403
No	442 (95.5)	10 (90.9)	432 (95.6)	
Yes	21 (4.5)	1 (9.1)	20 (4.4)	
Ever diagnosed with an STI				.490
No	449 (94.1)	10 (90.9)	439 (94.2)	
Yes	28 (5.9)	1 (9.1)	27 (5.8)	
Condom use frequency, past 4 months				**.003**
Never or hardly ever	109 (29.5)	8 (72.7)	101 (28.1)	
Sometimes, almost all the time or every time	261 (70.5)	3 (27.3)	258 (71.9)	
Ever perpetrated sexual violence				**.005**
No	413 (88.3)	6 (54.6)	407 (89.1)	
Yes	55 (11.7)	5 (45.5)	50 (10.9)	
Perpetrated sexual violence, past 4 months				**.026**
No	447 (93.7)	8 (72.7)	439 (94.2)	
Yes	30 (6.3)	3 (27.3)	27 (5.8)	
Binge drinking				.476
No	253 (53.0)	7 (63.6)	246 (52.8)	
Yes	224 (47.0)	4 (36.4)	220 (47.2)	
Drug use, past 30 days				.188
No	321 (67.7)	5 (45.5)	316 (68.3)	
Yes	153 (32.3)	6 (54.6)	147 (31.7)	
Smoking, past 30 days				.050
No	314 (66.2)	4 (36.4)	310 (67.0)	
Yes	160 (33.8)	7 (63.6)	153 (33.0)	
Military status				
No	457 (96.2)	9 (81.8)	448 (96.6)	.061
Yes	18 (3.8)	2 (18.2)	16 (3.5)	
Year in school				.613
1st year undergraduate	135 (28.4)	2 (18.2)	133 (28.6)	
2nd year undergraduate	113 (23.7)	5 (45.4)	108 (23.2)	
3rd year undergraduate	94 (19.8)	1 (9.1)	93 (20.0)	
4th year undergraduate	86 (18.1)	2 (18.2)	84 (18.1)	
5th year undergraduate	16 (3.4)	0	16 (3.4)	
Graduate or professional	27 (5.7)	1 (9.1)	26 (5.6)	
Other	5 (1.1)	0	5 (1.1)	
Athletic team member				.231
No	375 (78.8)	6 (60.0)	369 (79.2)	
Yes	101 (21.2)	4 (40.0)	97 (20.8)	
Greek life member				.406
No	396 (83.2)	8 (72.7)	388 (83.4)	
Yes	80 (16.8)	3 (27.3)	77 (16.6)	

Italics indicate *mean/SD*. Bold indicates significant at *p* < .05.

a By *t*-test, Chi-square or Fisher's exact test.

## Data Availability

Data available on request due to privacy/ethical restrictions
